# Virtual Reality–Based Treatment for Military Members and Veterans With Combat-Related Posttraumatic Stress Disorder: Protocol for a Multimodular Motion-Assisted Memory Desensitization and Reconsolidation Randomized Controlled Trial

**DOI:** 10.2196/20620

**Published:** 2020-10-29

**Authors:** Chelsea Jones, Lorraine Smith-MacDonald, Antonio Miguel-Cruz, Ashley Pike, Marieke van Gelderen, Liana Lentz, Maria Y Shiu, Emily Tang, Jeffrey Sawalha, Andrew Greenshaw, Shawn G Rhind, Xin Fang, Adrian Norbash, Rakesh Jetly, Eric Vermetten, Suzette Brémault-Phillips

**Affiliations:** 1 Heroes in Mind, Advocacy and Research Consortium Faculty of Rehabilitation University of Alberta Edmonton, AB Canada; 2 Department of Occupational Therapy Faculty of Rehabilitation University of Alberta Edmonton, AB Canada; 3 Glenrose Rehabilitation Hospital Research Innovation and Technology (GRRIT) Glenrose Rehabilitation Hospital Edmonton, AB Canada; 4 ARQ Centrum’45 Diemen Netherlands; 5 Department of Psychiatry Leiden University Medical Center Leiden Netherlands; 6 School of Health Studies Western University London, ON Canada; 7 Defence Research and Development Canada Toronto Research Centre Toronto, ON Canada; 8 Department of Psychiatry Faculty of Medicine and Dentistry University of Alberta Edmonton, AB Canada; 9 Department of Obstetrics and Gynecology Faculty of Medicine and Dentistry University of Alberta Edmonton, AB Canada; 10 Canadian Forces Health Services Department of National Defense Edmonton, AB Canada; 11 Department of Mental Health Canadian Forces Health Services Department of National Defense Ottawa, ON Canada; 12 Military Mental Health Research Ministry of Defense Utrecht Netherlands; 13 ARQ National Psychotrauma Centre Deimen Netherlands

**Keywords:** 3MDR, posttraumatic stress disorder, military, veteran, psychotherapy, virtual reality

## Abstract

**Background:**

Military members are at elevated risk of operational stress injuries, including posttraumatic stress disorder (PTSD) and moral injury. Although psychotherapy can reduce symptoms, some military members may experience treatment-resistant PTSD. Multimodular motion-assisted memory desensitization and reconsolidation (3MDR) has been introduced as a virtual reality (VR) intervention for military members with PTSD related to military service. The 3MDR intervention incorporates exposure therapy, psychotherapy, eye movement desensitization and reconsolidation, VR, supportive counselling, and treadmill walking.

**Objective:**

The objective of this study is to investigate whether 3MDR reduces PTSD symptoms among military members with combat-related treatment-resistant PTSD (TR-PTSD); examine the technology acceptance and usability of the Computer Assisted Rehabilitation ENvironment (CAREN) and 3MDR interventions by Canadian Armed Forces service members (CAF-SMs), veterans, 3MDR clinicians, and operators; and evaluate the impact on clinicians and operators of delivering 3MDR.

**Methods:**

This is a mixed-methods waitlist controlled crossover design randomized controlled trial. Participants include both CAF-SMs and veterans (N=40) aged 18-60 years with combat-related TR-PTSD (unsuccessful experience of at least 2 evidence-based trauma treatments). Participants will also include clinicians and operators (N=12) who have been trained in 3MDR and subsequently utilized this intervention with patients. CAF-SMs and veterans will receive 6 weekly 90-minute 3MDR sessions. Quantitative and qualitative data will be collected at baseline and at 1, 3, and 6 months postintervention. Quantitative data collection will include multiomic biomarkers (ie, blood and salivary proteomic and genomic profiles of neuroendocrine, immune-inflammatory mediators, and microRNA), eye tracking, electroencephalography, and physiological data. Data from outcome measures will capture self-reported symptoms of PTSD, moral injury, resilience, and technology acceptance and usability. Qualitative data will be collected from audiovisual recordings of 3MDR sessions and semistructured interviews. Data analysis will include univariate and multivariate approaches, and thematic analysis of treatment sessions and interviews. Machine learning analysis will be included to develop models for the prediction of diagnosis, symptom severity, and treatment outcomes.

**Results:**

This study commenced in April 2019 and is planned to conclude in April 2021. Study results will guide the further evolution and utilization of 3MDR for military members with TR-PTSD and will have utility in treating other trauma-affected populations.

**Conclusions:**

The goal of this study is to utilize qualitative and quantitative primary and secondary outcomes to provide evidence for the effectiveness and feasibility of 3MDR for treating CAF-SMs and veterans with combat-related TR-PTSD. The results will inform a full-scale clinical trial and stimulate development and adaptation of the protocol to mobile VR apps in supervised clinical settings. This study will add to knowledge of the clinical effectiveness of 3MDR, and provide the first comprehensive analysis of biomarkers, technology acceptance and usability, moral injury, resilience, and the experience of clinicians and operators delivering 3MDR.

**Trial Registration:**

ISRCTN Registry 11264368; http://www.isrctn.com/ISRCTN11264368.

**International Registered Report Identifier (IRRID):**

DERR1-10.2196/20620

## Introduction

### Background

Posttraumatic stress disorder (PTSD), a mental health condition triggered by experiencing or witnessing a terrifying event, can be life-altering. Characterized by enduring symptoms related to negative cognitive intrusions, avoidance, hypervigilance, and alterations in mood, arousal, and reactivity [1], PTSD is the most common mental illness experienced by military members and veterans [2-4], and remains the predominant focus of most military and veteran health research and care [5-8]. PTSD prevalence is persistent and may increase over time [9], characterized by complexity and a wide variance internationally within military and veteran populations [10]. Among US military members deployed during the War on Terror, PTSD prevalence estimates reach up to 19% [10] compared to 5.3% for Canadians [11], 2.7% to 4% in UK military members [12], and 3% for military members from the Netherlands [13]. A recent meta-analysis reported that overall rates remained high (at approximately 23%) for US veterans following 9-11 [14] and the incidence of PTSD increased to 16% for Canadian veterans [15].

Isolated or cumulative traumatic experiences can also cause long-term psychological and spiritual struggles, including depression, anxiety, moral injury [5,16], and suicide [17]. Moral injury—a separate trauma syndrome that results from exposure to ethically or virtuously injurious experiences such as witnessing or participating in acts that transgress personal morals and values [18]—has been posited as a potential key comorbidity, and a compounding and complicating factor of PTSD [19,20].

The classification of treatment-resistant PTSD (TR-PTSD) has been adopted for the many veterans who do not experience a clinically significant reduction in symptoms following receipt of evidence-based treatment. International PTSD guidelines consistently demonstrate trauma-focused cognitive behavioral therapy, cognitive processing therapy, prolonged exposure, and eye-movement desensitization and reprocessing (EMDR) to be the gold-standard and first-line treatments for PTSD. However, it is equally acknowledged that military members and veterans consistently have poorer clinical outcomes than their civilian counterparts in these treatments [21-23]. Although general recommendations for optimizing psychotherapies (ie, using untried or complementary modalities) and utilizing secondary pharmacological interventions (ie, mood stabilizers, antiadrenergic agents/hypnotics, or atypical antipsychotic agents) have been suggested [24], specific evidence-based TR-PTSD therapies or protocols are lacking.

### Multimodular Motion-Assisted Memory Desensitization and Reconsolidation

**Figure 1:**
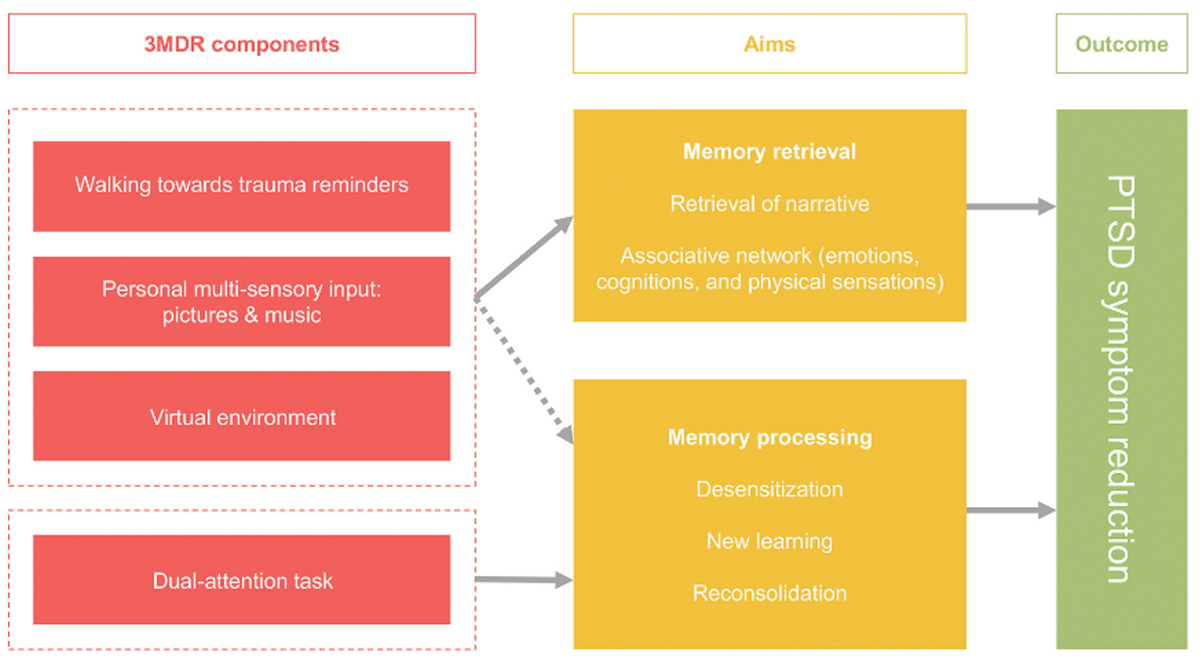
Schematic overview of the augmentation strategies applied in the framework of the multimodular motion-assisted memory desensitization and reconsolidation (3MDR) intervention and its outcomes. Used with permission [25]. PTSD: posttraumatic stress disorder.

Multimodular motion-assisted memory desensitization and reconsolidation (3MDR) is an emerging virtual reality (VR)-assisted therapy being studied with military members and veterans in the Netherlands, United States, United Kingdom, and Canada. It is hypothesized that the combination of trauma modalities in 3MDR may contribute to its potential effectiveness. The key elements of 3MDR therapy include: (1) engaging in exposure to traumatic material whereby avoidance patterns are minimized or broken; (2) experiencing emotions in the here and now that are evoked while the traumatic memories are being retrieved, and giving expression to these; and (3) reintegrating (reconsolidating) memories and sensory along with affective information associated with the trauma via a dual task (Figure 1) [25,26]. It is also thought that the combination of elements involved in 3MDR work collectively to facilitate the memory reconsolidation necessary to reduce the intensity of traumatic memories and subsequent PTSD symptoms. These include (1) exposure to VR visual imagery and auditory input, (2) walking, (3) a dual-attention task, and (4) therapeutic context and relationship [25,26]. A brief description of each element follows.

#### Exposure to Visual and Auditory Input

Emotional dysregulation and numbing are increasingly being considered as crucial components to address in PTSD treatment, especially for veterans who appear to more actively withhold emotional responses and use numbing as a coping mechanism [27]. Viewing affective pictures is thought to increase emotional engagement, and has been found to elicit physiological response patterns, which are higher for unpleasant pictures [28,29]. However, in addition to looking at affective pictures [30], auditory input such as music is noted to be a strong emotional trigger [31]. As self-selected music can elicit the retrieval of significantly more autobiographical memories in people with dementia [32,33], it is considered that optimal memory recollection may likewise be elicited by music for those with PTSD. Patients diagnosed with PTSD have indicated that music evokes (traumatic) memories, and enables them to access and discuss these memories [25,34,35]. Visual and auditory components are therefore central to 3MDR because of their believed ability to evoke potentially repressed or numbed emotions.

#### Walking

Among those with PTSD, walking toward an image of a traumatic memory has been found to increase engagement, decrease avoidance [36], potentially support the formation of new cognitive and emotional experiences related to the traumatic event, and contribute to reconsolidation of the traumatic memory [25]. Walking seems to facilitate consolidation of fear-related and traumatic memories. This occurs through bilateral movement [25], associated increases in brain-derived neurotrophic factor levels [37], approach behaviors toward feared cues and challenging working memory [33], increasing expression of associative memory [38], enhancing divergent thinking (which facilitates accessing emotional and cognitive networks associated with the traumatic memory) [37,39], allowing for new memories to surface [39], and reducing somatic and dissociative symptoms [40]. Walking throughout 3MDR’s platform phase is thought to be foundational to the therapy.

#### Dual-Attention Task

The dual-attention task component of 3MDR aims to facilitate processing and reconsolidation of traumatic memories and utilizes aspects of EMDR [31,41,42]. In traditional EMDR interventions, participants are asked to retrieve aversive memories and rate their vividness and emotional intensity [41-45]. They are then asked to recall the memories while making eye movements or performing a bilaterally stimulating task [41-45]. Within the 3MDR intervention, this task requires the participant to visually track a horizontally moving ball across the screen in the foreground of a traumatic image while calling out numbers displayed on this ball. Reconsolidation theory posits that a memory, while it is being recalled, becomes pliable for some time in which new information may be added [25,43]. Working memory is considered to be taxed when the brain is required to simultaneously perform two tasks. The first task (ie, viewing a traumatic picture) receives less attention and attenuates the vividness and emotional tone of the memory [44-46]. While participants are recalling traumatic memories in the course of 3MDR, more context or new information can be consolidated so that memories are not experienced as a current threat or as emotionally charged [25].

### Therapeutic Context and Relationship

The therapeutic setting of 3MDR is an active side-by-side rather than passive position, which impacts both the physical context and therapeutic alliance. The patient is supported throughout the process by a therapist who is standing alongside, and, more importantly, is also viewing the same traumatic material while the patient is recalling their traumatic event. This emotional participation in treatment is increased by the psychotherapeutic approach where the therapist asks the patient to describe the image, and their memory, somatic responses, cognitions, and emotions. Lower emotional valence and processing of the traumatic memories is expected, with new learning and the full traumatic network activated along with intervening in the reconsolidation process [25].

Findings from a 3MDR proof-of-concept study [40] and two initial randomized controlled trials (RCTs) [26,47] involving military members and veterans with combat-related TR-PTSD have been promising. In the published RCTs, decreases in PTSD symptom severity from baseline to the trial end point were significantly greater for the 3MDR group as compared to those of the control group with medium to large effect sizes [26,47]. Notably, these effects were maintained for weeks after the conclusion of the 3MDR intervention and a low dropout rate was noted in both studies. Although the results of the primary outcome, a reduction in PTSD symptoms, were favorable, variable results in the secondary outcomes were obtained, and many questions about 3MDR remain, warranting further examination.

### Overall Aims

This protocol describes a novel study to assist with the knowledge translation of the underpinnings of 3MDR as a therapeutic intervention. The primary study aim is to investigate whether the 3MDR intervention reduces symptoms of combat-related TR-PTSD and moral injury compared with treatment as usual among Canadian Armed Forces service members (CAF-SMs) and veterans. The study will be the first of its kind to examine the technology acceptance and usability of the Computer-Assisted Rehabilitation ENvironment (CAREN) VR system for 3MDR by participants, 3MDR clinicians and operators, as well as the impact of delivering 3MDR on clinicians and operators. Further, the longitudinal nature of the study will facilitate analysis of the longevity of possible 3MDR treatment effects for up to 6 months postintervention. This study will also address the possible effect of 3MDR on the constructs of moral injury and resilience. Further, as examination of relationships of psychological and biological factors to effective PTSD treatment has become critically important in attempts to understand the pathogenesis of TR-PTSD and to identify novel therapeutic targets [48-50], biomarker measurements and machine learning will be employed. Classifying neuropsychiatric disorders on the basis of objective and practical biomarkers aims to inform the diagnosis and selection of therapeutic approaches [51-54]. Machine-learning analysis will attempt to develop predictive models that can be used for individual diagnosis and prediction of the course of illness and effectiveness of treatment. Biomarker and machine learning utilization is a unique and novel addition to the study of 3MDR that has yet to be incorporated into other 3MDR studies internationally.

The main research questions to be addressed include: (1) Will participants’ combat-related TR-PTSD and moral injury symptoms, and associated behavior and physiology, change after an intervention of 3DMR therapy compared with treatment as usual? (2) Will peripheral multiomic biomarkers related to traumatic stress (ie, blood and salivary proteomic and genomic profiles of neuroendocrine, immune-inflammatory responses, and microRNA) be modified by the 3MDR intervention and show utility for predicting patient outcomes posttreatment? (3) What are the 3MDR clinicians’, operators’, and participants’ perceived technology acceptance and usability of 3MDR on the CAREN system? (4) What is the overall participant experience of the 3MDR intervention and comparability to PTSD therapies that the participants had previously engaged in? (5) Will 3MDR clinicians and operators experience perceived or secondary traumatic stress, or a change in their professional quality of life as a result of delivering 3MDR?

### Specific Objectives

The first objective of the study is to examine the effect of 3MDR therapy on combat-related TR-PTSD and moral injury symptoms, behaviors, and physiological measures for CAF-SMs and veterans who have not benefited from other trauma therapies. The second objective is to examine the perceived technological acceptance and usability of 3MDR by CAF-SMs and veterans with combat-related TR-PTSD as well as 3MDR clinicians and operators. The third objective is to examine the effects of 3MDR on the operators and clinicians who provide this intervention to military members and veterans with combat-related TR-PTSD. In addition to conventional statistical analyses, the final objective of this study is to explore the application of machine learning in an attempt to identify individual data patterns that may predict diagnosis, severity, and potential treatment outcomes.

### Hypotheses

Related to the objectives above, we have derived the following four hypotheses for this study.

Hypothesis 1: Participants’ symptoms of combat-related TR-PTSD will decrease during the 3DMR therapy compared with treatment as usual.

Hypothesis 2: CAF-SMs, veterans, 3MDR clinicians, and operators will perceive 3MDR to be a novel intervention with adequate perceived technology acceptance and usability.

Hypothesis 3: 3MDR operators and clinicians will not experience adverse mental health effects, including secondary trauma and stress, that affect their daily functioning as a result of 3MDR delivery.

Hypothesis 4: Machine-learning analysis will generate two accurate predictive models that may portend diagnosis, severity, and potential treatment outcomes in CAF-SMs and veterans with combat-related TR-PTSD.

## Methods

### Intervention

**Figure 2:**
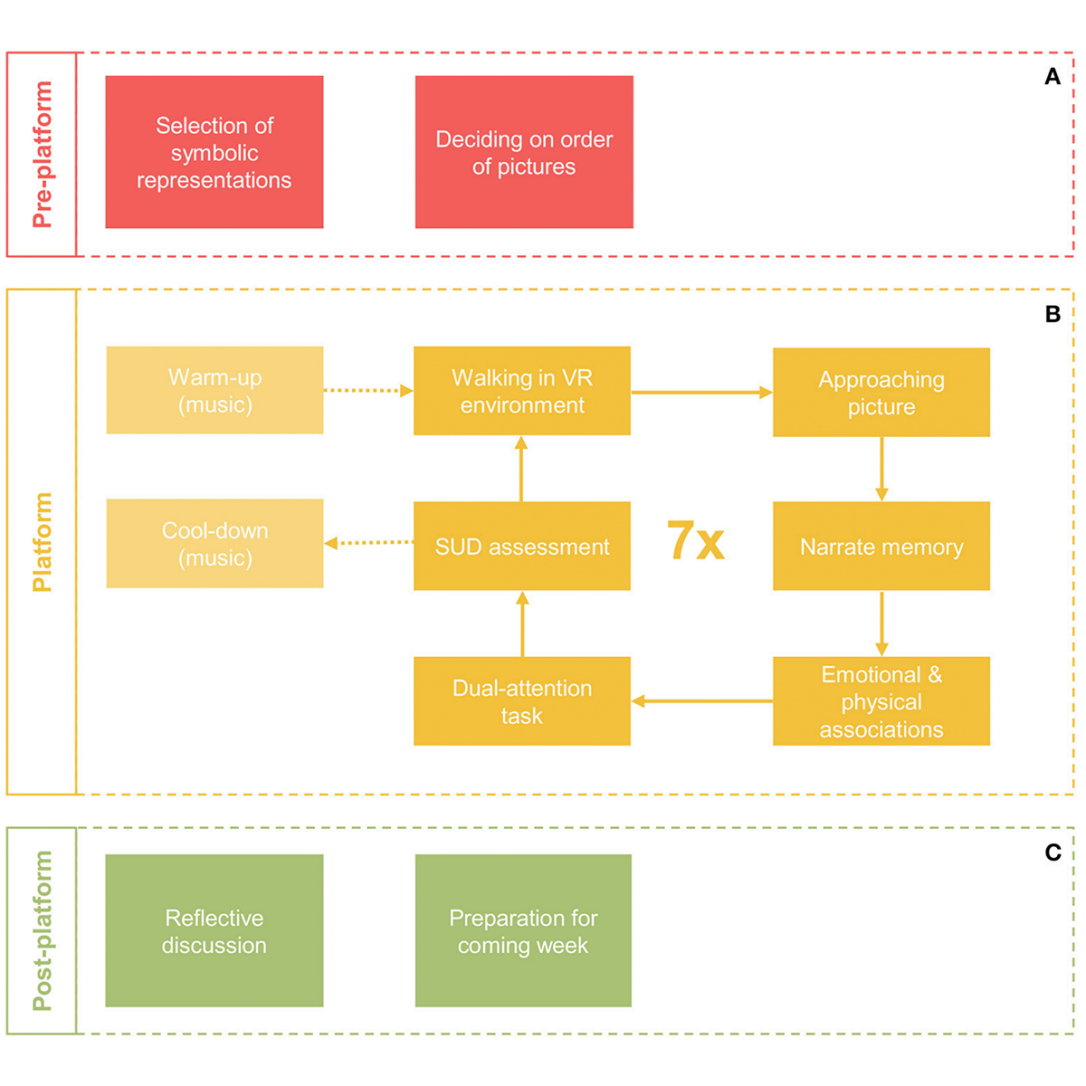
Schematic overview of a single multimodular motion-assisted memory desensitization and reconsolidation (3MDR) session. A session consists of a preplatform phase (A), platform phase (B), and postplatform phase (C). Used with permission [25]. VR: virtual reality; SUD: Subjective Units of Distress scale.

The 3MDR intervention takes place in an immersive CAREN, a room-sized, three-dimensional VR system that facilitates full engagement. A treadmill located in a central platform in the CAREN is surrounded by 240° floor-to-ceiling screens with motion-capture technology. In each session, the participant continually walks on the treadmill while briefly listening to self-selected music reminiscent of their military deployment(s) prior to viewing a series of 7 self-selected images related to their traumatic events projected onto the screen. The participant is directed to engage in their selected image and walk toward it. Each 3MDR session follows a repetitive cycle of a preplatform phase (A), which is a mental and physical warm up; a platform phase (B) when the VR environment changes, providing a visual and auditory cue of movement into cycles of active therapy; and, following a mental cool-down period, a postplatform phase (C), during which the participant and clinician reflect on the session, and focus on important or newly arisen cognitive and emotional associations (Figure 2). A self-care plan is also reviewed prior to the participant leaving the CAREN. A typical 3MDR session lasts 90 minutes, including the participant walking on the platform for 45-60 minutes (equivalent to 3-4 km).

In the context of the full-service hospital setting, a risk mitigation strategy will be implemented. Clinicians providing 3MDR who have vast experience with trauma-affected populations, including CAF-SMs and veterans, will be authorized to provide mental health services. Clinicians and operators will continually assess the safety of participants during sessions, and will stop the intervention and implement codes or safety protocols if required. Participants will be debriefed after therapy to determine if they are safe to leave the site and prepared to follow their self-care and safety plan. Alternate participant transportation will be utilized for participants if required.

### Study Design and Setting

This study will be a nonblinded mixed-methods waitlist controlled staggered entry RCT with a crossover design. An experimental group (n=20) will receive 6 sessions of 3MDR once a week followed by treatment as usual. The definition of treatment as usual for the purpose of this study will include other evidence-based psychotherapeutic interventions such as cognitive behavioral therapy, cognitive processing therapy, prolonged exposure, EMDR, and medications.

A waitlist control group (n=20) will initially receive treatment as usual for 9 weeks before being offered the opportunity to receive the 3MDR intervention. Those agreeable to the 3MDR intervention will then receive 6 sessions of 3MDR before resuming treatment as usual. Assignment to the initial experimental group versus the waitlist control group will be randomized. Participants will receive an introductory session prior to commencement of the 3DMR intervention and a follow-up session after completion of the intervention. All participants will provide informed written consent. For the projected timeline of this study, refer to Table 1.

This study has been approved by the University of Alberta Research Ethics Board (Pro00084466) and Canadian Armed Forces Surgeon General (E2019-02-250-003-0003).

**Table 1:** Time points of data collection for participants in the intervention group (1) and control group (2).

Construct to be measured	Measure	Week 1 (Baseline)	Week 3	Weeks 4-8	Week 9 (Post)	1 Month Post	3 Months Post	6 Months Post	12 Months Post
PTSD^a^	LEC5^b^	1, 2							
PTSD	CAPS5^c^	1, 2			1, 2		1, 2	1, 2	1
PTSD	PCL-5^d^		1	1	1, 2	1, 2	1, 2	1, 2	1
Demographic Information	Questionnaire	2	1						
Anxiety	GAD-7^e^		1	1	1, 2	1, 2	1, 2	1, 2	1
Depression	PHQ-9^f^		1	1	1, 2	1, 2	1, 2	1, 2	1
Avoidance	PABQ^g^		1	1	1, 2	1, 2	1, 2	1, 2	1
Dissociation	PDEQ^h^		1	1	1, 2	1, 2	1, 2	1, 2	1
Alcohol Use	AUDIT^i^		1	1	1, 2	1, 2	1, 2	1, 2	1
Moral Injury	MISS-M^j^		1	1	1, 2	1, 2	1, 2	1, 2	1
Emotional Regulation	DERS-18^k^		1	1	1, 2	1, 2	1, 2	1, 2	1
Resilience	CD RISC-25^l^		1	1	1, 2	1, 2	1, 2	1, 2	1
Social Functioning	OQ-45^m^		1	1	1, 2	1, 2	1, 2	1, 2	1
Quality of Life	EQ-5D^n^/EQ VAS^o^		1	1	1, 2	1, 2	1, 2	1, 2	1
Neurofunctional Performance	BrainFX Screen	1, 2			1, 2				
Technology Acceptance and Usability	UTAUT^p^ Questionnaire		1		1				
Subjective Distress	SUDS^q^	1, 2	1	1	1, 2	1, 2	1, 2	1, 2	1
Client Satisfaction	CSQ-8^r^			1	1				
3MDR^s^ Satisfaction	3MDR-Q^t^			1	1				
3MDR Satisfaction	Subjective Interview		1	1	1	1	1	1	1

^a^PTSD: posttraumatic stress disorder.

^b^LEC5: Life Events Checklist for DSM-5.

^c^CAPS5: Clinically Administered PTSD Scale for DSM-5.

^d^PCL-5: PTSD Checklist for DSM-5.

^e^GAD-7: Generalized Anxiety Disorder Scale, 7-item.

^f^PHQ-9: Patient Health Questionnaire, 9-item.

^g^PABQ: Posttraumatic Avoidance Behavior Questionnaire.

^h^PDEQ: Dissociative Experiences Questionnaire.

^i^AUDIT: Alcohol Use Disorder Identification Test.

^j^MISS-M: Moral Injury Symptom Scale for Military.

^k^DERS-18: Difficulties in Emotional Regulation Scale.

^l^CD RISC-25: Connor-Davidson Resilience Scale.

^m^OQ-45: Outcome Questionnaire 45.

^n^EQ-5D: EuroQol-5D-5L.

^o^EQVAS: EuroQol-Visual Analog Scale.

^p^UTAUT: Unified Theory of Acceptance and Usability of Technology.

^q^SUDS: Subjective Units of Distress Scale.

^r^CSQ-8: Client Satisfaction Questionnaire.

^s^3MDR: multimodular motion-assisted memory desensitization and reconsolidation.

^t^3MDR-Q: multimodular motion-assisted memory desensitization and reconsolidation questionnaire.

### Recruitment

Regular and reserve CAF-SMs and veterans will be recruited through established relationships with the CAF, Operational Stress Injury Clinics, the Royal Canadian Legion Alberta/Northwest Territories Command, and local community service providers supporting CAF-SMs and veterans. Participants will also be recruited through word of mouth among potential participants. Eligible participants include English-speaking regular and reserve CAF-SMs and veterans aged 18-60 years who meet the Diagnostic and Statistical Manual-5 (DSM-5) criteria for a diagnosis of PTSD. This includes: (1) symptoms lasting more than 3 months; (2) a score of 30 or higher on the DSM-5 Clinician-Administered PTSD Scale, Fifth Edition (CAPS-5) interview; (3) trauma related to combat experiences; and (4) have had a nonresponse to at least two types of evidence-based PTSD treatments where at least one of these treatments was a psychotherapeutic intervention. It is permitted that the second treatment is a pharmacological intervention. Participants must also be stable on their psychotropic medication for a period of 4 weeks before entering the trial and agree to notify researchers of medication adjustments during the course of the trial. Participants with comorbidities will be included if they satisfy the other criteria and PTSD is considered the primary diagnosis. Participants must also have the cognitive capacity to provide informed consent. Participants will be excluded from the study if they demonstrate signs of acute suicidality, psychosis, or reduced cognitive processing that would exclude them from following directions or providing informed consent. Participants will also be excluded if it is determined that they are unable to walk at a normal pace for 30-45 minutes on a treadmill, or if their physical size or abilities are not compatible with the CAREN system. For CAF-SMs and veterans, screening by a research team member will consist of an interview to discuss their previous military employment/deployments, current and past medical history, history and experiences of previous PTSD interventions, and overall suitability for the study.

Recruitment of 3MDR clinicians and operators (N=12) will be conducted through word of mouth among the six international 3MDR research teams located in the Netherlands, the United States (California and Maryland), the United Kingdom (Wales), and Canada (Alberta and Ontario). Clinicians and operators who have received training for 3MDR and completed 6 weeks of interventions will be eligible for a pre/post evaluation. Voluntary written informed consent will be obtained from all participants.

The control group will undergo screening for eligibility and baseline assessment, followed by 9 weeks of treatment as usual while the intervention group receives the 3MDR intervention. They will then be provided an opportunity to cross over into the intervention group.

For the introductory session, intervention group participants will become accustomed to the CAREN system by practicing the process associated with 3MDR, including walking on the treadmill while looking at a neutral image as the clinician guides them through the 3MDR protocol. The participant will be asked to provide (1) images reminiscent of the traumatic deployment event that preceded a PTSD diagnosis, ranked according to the emotional distress that each elicits (ie, from least to greatest level of distress); and (2) digital music tracks of a song that reminds them of their deployment and other sounds or music that provide positive feelings. A secondary purpose of this introductory session is also to transfer trust from their service provider to the 3MDR researchers and clinicians. One week after the introductory session, the 3MDR therapy will be initiated and participants will undergo 6 90-minute sessions.

### Randomization and Blinding

Participants will be randomized to the 3MDR or treatment as usual groups in a parallel design. A research assistant will utilize computerized random number allocation to assign participants to either the 3MDR or treatment as usual groups and maintain a confidential log of which participants are assigned to the respective groups. In addition, participants will be randomly assigned to the clinician that they will be engaging with for 3MDR. Participants, assessors, 3MDR clinicians, and other health care providers will not be blinded to the group allocation.

### Sample Size

A sample size calculation was conducted using G*Power 3 [55], based on the primary hypothesis that 3MDR would result in significantly lower clinician-rated PTSD symptoms as compared to treatment as usual. To detect a significant interaction with at least a medium effect size, a minimal sample size of 17 participants was determined for each group. Because of an estimated 10% dropout and some attrition at measurements, the sample size was set at 20 participants in each group (Cohen f=1.0) using general linear modeling with one within-subjects factor (2 time points) and one between-subjects factor (2 interventions), and assuming a correlation of r=0.5 between the repeated measures, an α-level of .05, and a power (1 – β) of 0.8.

### Study Timeline and Data Collection

#### Objective 1: Effectiveness of 3MDR

**Figure 3:**
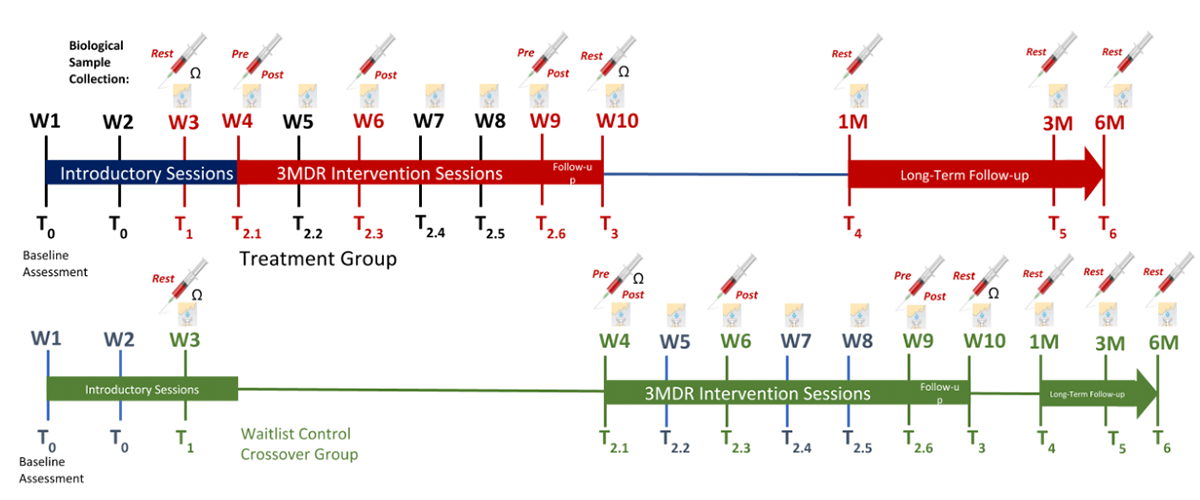
Multimodular motion-assisted memory desensitization and reconsolidation (3MDR) data collection time points for the intervention (treatment; blue/red) and waitlist control (green) groups. The syringe icon denotes blood collection and the square icon denotes saliva collection.

Data will be collected from intervention and control group participants at initial assessment (T0), prior to delivery of 3MDR (T1), pre/post 3MDR sessions (T2.1-2.6), and at 1 week (T3), 1 month (T4), 3 months (T5), and 6 months (T6) postintervention (Figure 3).

Clinical outcome measures will be collected at each time point using the questionnaires listed in Table 1. Qualitative interviews will occur at T3 through T6. Physiological, biometric, and qualitative audio/video data will be collected at T2.1-T2.6. Blood and saliva samples will be collected at baseline (T1), before (pre) and immediately after (post) 3MDR sessions at T2.1 and T2.6, post 3MDR sessions at T2.3 and T2.4, at 1-week follow up (T3), and at 1-, 3-, and 6-month follow ups (T4, T5, T6) for the analyses of peripheral blood and salivary biomarkers, including inflammatory mediators, neuroendocrine hormones, and microRNAs (see Multimedia Appendix 1).

Demographic information will include gender, age, ethnicity, marital status, military branch (ie, army, navy, or air force), and occupation. Psychological outcomes will be assessed through paper-based questionnaires administered during each contact with participants (Table 1). For further information on each outcome measure, refer to Multimedia Appendix 2.

##### Physiological Data

Walking and eye-scanning patterns, as well as physiological data (eg, heart rate, breathing rate, gait pattern, force plate analysis) will be collected during the training session through the CAREN system, Tobii Mobile eye-tracking glasses, Muse electroencephalogram (EEG), and Zephyr BioHarness 3.

##### Subjective Measures

Video recording of each session will capture qualitative data of the exchange between the clinician and participant during the 3MDR session. Audio recordings of the therapeutic debriefs will also occur, during which the clinician and patient will discuss the experience of any remarkable or meaningful aspects of the session. The clinician will also share observations. Upon completion of the 3MDR intervention, audio-recorded iterative semistructured interviews will be conducted with all participants at week 10, and 1, 3, and 6 months postintervention to explore their experience of 3MDR, its impact on their PTSD symptoms and overall function, and participation in the research study.

#### Objective 2: Measure of Technology Acceptance and Usability

To measure the perceived technology acceptance and usability of 3MDR and the CAREN, two 15-question surveys based on the Unified Theory of Acceptance and Usability of Technology (UTAUT) model [56] will be administered pre/post exposure to CAREN and 3MDR. The surveys utilized Likert scores ranging from 15 to 105 points, with high scores indicating increased perceived technology acceptance and usability.

#### Objective 3: 3MDR Operators and Clinicians

The impact of delivering 3MDR will be examined with clinicians and operators using questionnaires related to technological acceptance, perceived stress, and professional quality of life prior to 3MDR training and on completion of a full course of 3MDR with a study participant (Table 2).

**Table 2:** Time points of data collection for multimodular motion-assisted memory desensitization and reconsolidation (3MDR) clinicians and operators.

Construct to be measured	Measure	Week 1 (Baseline)	Week 9 (Post)
Technology Acceptance and Usability	UTAUT^a^ Questionnaire	X	X
Perceived Stress	PSS^b^	X	X
	SUDS^c^	X	X
	Subjective Interview		X
Professional Quality of Life	PQoL^d^	X	X
Secondary Stress	STSS^e^	X	X

^a^UTAUT: Unified Theory of Acceptance and Usability of Technology.

^b^PSS: Perceived Stress Scale.

^c^SUDS: Subjective Units of Distress Scale.

^d^PQoL: Professional Quality of Life Scale.

^e^STSS: Secondary Traumatic Stress Scale.

#### Objective 4: Machine Learning

Although group-based statistical analysis is useful for drawing conclusions about general trends, it has limited utility for guiding individual diagnostic and treatment choices.

As a result, in addition to conventional statistical analysis, this study will explore the application of machine learning in an attempt to identify individual data patterns that may predict diagnosis, severity, and potential treatment outcomes. Physiological, biometric, biological, and qualitative data from T0 to T3 will be used to predict psychological outcome measures at week 9 plus follow-up sessions. A model that can compute sequential, multimodal data is needed to autonomously observe and learn the variability of the relevant feature sets and its association with outcome measures [57,58]. Deep neural networks will be applied for this task [57,58].

### Data Analysis

#### Objective 1: Effectiveness of 3MDR

The quantitative analysis strategy for this study will include a variety of descriptive, parametric, nonparametric, univariate, and multivariate approaches, as well as machine-learning techniques.

Descriptive statistics will be used to summarize demographic data of participants, including mean values, frequencies, and proportions.

Baseline between-group differences in sociodemographic, clinical, and neuropsychological variables will be assessed. For psychological outcomes, including self-reported PTSD symptom severity, anxiety and depression symptoms, and avoidance behavior, analyses will be conducted using mixed linear models. All of the analyses above will be carried out on an intent-to-treat basis. A two-tailed *P*<.05 will be considered statistically significant.

For preliminary assessment of the relation between treatment outcomes and relevant predicting

variables, exploratory regression analyses will be performed and a predicting variable will be considered to have potential predicting value in cases of *P*<.20. Predictors will then be combined in a regression model to determine which variables most strongly predict 3MDR treatment success. Missing data points, potentially due to missed visits or outcome measure abnormalities, will be handled with the last observation carried forward analysis of covariance [59].

A partial least squares (PLS) regression [60] will be used to identify combinations of blood biomarker concentrations that may distinguish the three treatment groups. PLS is a multivariate data reduction technique that creates orthogonal latent variables describing the maximal covariance between a set of predictors (biomarkers) and response (patient outcome) variables [61]. Between-group comparisons will be evaluated post hoc by generating bootstrapped mean differences of the group saliences for all group pairs, followed by the derivation of bootstrap ratios and empirical *P* values. In addition, model prediction performance will be quantitated by evaluating the prediction accuracy and posterior probability from separate PLS-discriminant analyses (PLSDA) on each group pair. PLSDA will also be used to evaluate the differences in biomarker profiles between groups. For both tasks, PLS and PLSDA, the weighted contribution of individual biomarker loadings will be quantitated by bootstrapped resampling (5000 iterations), followed by the generation of bootstrapped ratios and empirical *P* values. For evaluation of correlations between blood biomarkers and patient outcomes, an out-of-sample, leave-two-out cross-correlation (R2) value will be calculated on the PLS model. Random forest–based feature selection will be used to select the most relevant biomarkers that could be used to differentiate treatment groups. The random forest machine-learning algorithm is robust, with low bias and a reduced chance for overfitting.

For qualitative data analysis, audio recordings will be professionally transcribed and thematically analyzed in NVivo 12 software [62]. Thematic analysis involves examining text in detail to identify recurring patterns (“themes”) through both inductive and deductive reasoning [63]. Open codes will be combined into preliminary patterns that focus on similarities and differences within and between audio recordings to reduce the number of codes and provide more focus for the secondary level of coding [63]. Within the secondary level of coding, more abstract concepts will be assigned to broader categories of themes, and the relations between these concepts will be explored and verified through key quotes. Results will be distributed to a sample of the participants for member checking to assure the accuracy and trustworthiness of the research team’s interpretation of themes.

#### Objective 2: Measure of Technology Acceptance and Usability

The UTAUT questionnaires measure performance expectancy, effort expectancy, social influence, and facilitating conditions, and behavioral intention with respect to 3MDR and the CAREN [56]. Differences in the medians between groups (ie, performance expectancy, effort expectancy, social influence, and facilitating conditions, and the behavioral intention responses of intervention and control groups) will be analyzed by nonparametric tests. Acceptance will be assessed using the PLS structural equation model to determine reliability measurements for each construct (Cronbach α).

#### Objective 3: 3MDR Operators and Clinicians

Owing to the small anticipated sample size, nonparametric statistical analysis with the Wilcoxon signed-rank test will be employed to assess the outcome measures addressing clinician and operator stress. Qualitative interviews will be thematically analyzed as described above.

#### Objective 4: Machine Learning

In a supervised regression learning approach, the input of the machine-learning pipeline is considered to be the various types of data collected for each individual across every session. If the performance task is predicting reductions in symptom severity, then the output will contain psychological outcome measures. Such data will be divided into training and testing examples, whereby the statistical model will learn on the training set and then make data-driven predictions on the testing set to gauge how accurate those predictions were. During this process, the learner will optimize its predictions by comparing the predicted output with the observed output and then updating the parameters based on the severity of the errors made. Ultimately, the learned model will make individualized predictions about how a patient may respond to the treatment of 3MDR at a given time point.

### Triangulation of Data

A concurrent parallel approach following a data transformation model will be utilized in the data analysis process to converge the data for comparing and contrasting the quantitative statistical results with qualitative findings [64,65]. Integration will be scheduled at approximately every 5 participants during the data collection period to assist with the assurance of clinical appropriateness for the participants of the study and provide preliminary results. The final point of integration and analysis will take place at the conclusion of data collection.

## Results

The trial is currently underway with 11 CAF-SMs/veterans having completed the intervention protocol. Additionally, 5 clinicians and 4 operators have completed pre/post outcome measures or the interview component of the trial. Data will be analyzed with the goal of publication at the completion of the study within 2 years.

## Discussion

### Projected Outcomes

Effective evidence-based interventions are needed for the treatment of TR-PTSD experienced by military members and veterans. This 3MDR study, one of the first international studies of its kind, will investigate whether 3MDR reduces symptoms of combat-related TR-PTSD. This 3MDR study protocol has roots in previous 3MDR studies from Canada, the Netherlands, and the United Kingdom [25,40,48,66]. Ideally, the combined international efforts of multiple research teams could lead to a meta-analysis of 3MDR in the military and veteran populations. If 3MDR is found to be effective, study findings will support making 3MDR more accessible nationally and internationally as an intervention for CAF-SMs and veterans. If indicated, 3MDR trials could be extended to other trauma-affected populations (eg, public safety personnel, marginalized groups, some indigenous communities, victims of violent crime, immigrants, refugees, victims of natural disasters) with PTSD and various mental health conditions.

Although some alterations and additions have been incorporated in this study that make it distinct from other international studies, an effort has also been made to standardize various components across all studies. This includes consistency with 3MDR clinician and operator training, a 3MDR manual as a standardized intervention, 3MDR fidelity checklists, use of similar outcome measures where appropriate, and standardization of mixed-methods data analysis wherever possible.

This Canadian 3MDR study is the first to include a number of novel components. Uniquely, this longitudinal study will address resilience, moral injury, inflammatory mediators, neuroendocrine hormones, and microRNA via measurements of blood-based biomarkers. Collection of physiological data using novel technology such as eye tracking and EEG is also unique and will provide valuable insight into the mechanisms by which 3MDR may be effective. Expanding the knowledge of the efficacy of psychological treatments such as 3MDR specific to combat-related TR-PTSD is unique, as this population is rarely included in clinical trials [67].

The 3MDR intervention itself will be delivered by members of a multidisciplinary team of registered trauma-informed clinicians (ie, psychologists, social workers, occupational therapists, CAF mental health chaplains, psychiatrists) who have variable experience working with military members and veterans. The study will also examine secondary stress that may be experienced by clinicians and operators as a result of delivering 3MDR. Further, the acceptance and usability of technology by participants, and 3MDR clinicians and operators regarding the use of the CAREN for delivery of 3MDR will be studied.

It is predicted that a novel and synergistic effect between components of the 3MDR intervention and treatment outcomes for combat-related TR-PTSD will be apparent. This project will expand knowledge of the underlying mechanisms of TR-PTSD. Additionally, through machine learning, successful model development may predict changes in PTSD symptom severity in this context [68]. It is also hoped that prognostic models will be developed based on the study data from this protocol [61]. These models will enable clinical decisions—based on cognitive, biological, and psychological markers and symptom severity—to signal treatment responsiveness at the individual therapy client level [67].

The importance of determining effective components of 3MDR has value not only for this modality but also more broadly regarding PTSD treatment effectiveness. As veterans seem to gain limited progress in traditional trauma modalities, it may be that combat-related trauma necessitates the engagement of multiple biological, cognitive, and affective systems simultaneously to produce the effects found in civilian populations. If the combination of multiple efficacious components (eg, bilateral movement, dual-task attention, emotional engagement, visual and auditory stimulation) is effective at reducing PTSD symptoms, significant evaluation of the means by which currently accepted PTSD treatment modalities are delivered, most often including office environments and stationary physical positions, is warranted.

Moreover, if moral injury is also identified as a pertinent comorbid component of combat-related TR-PTSD, key insights may be gained as to why military members and veterans did not have an adequate response to previous treatments. Although research on moral injury is still burgeoning, this study has significant potential to further elucidate the overlap and difference between PTSD and moral injury, particularly in relation to Criterion D of the PTSD diagnosis [1]. Equally, as evidence-based interventions are currently very limited for moral injury, using 3MDR to treat moral injury may have a unique advantage as comorbid PTSD pathologies could be concurrently addressed, thus not requiring military members and veterans to undergo multiple psychotherapeutic interventions. The question of resilience is also relevant to the broader conversation, particularly if once the acute PTSD and moral injury symptoms are addressed, military members and veterans experience a sense of resilience in relation to any remaining pathology, social engagement, and improved quality of life.

### Limitations

Despite the many strengths of this study, some limitations must be noted. First, the limited intervention timeline (ie, a total of 6 sessions) may not be sufficient for changes to occur, particularly within participants who have had combat-related TR-PTSD for a significant period of time; however, similar studies for the treatment of combat-related TR-PTSD using 3MDR have shown positive and statistically significant changes in PTSD symptoms [25,47]. Second, it is unlikely that the results will apply to the general population for PTSD; larger clinical trials will be necessary for generalization as the intervention to date has been exclusively piloted with military members and veterans with combat-related TR-PTSD. Third, it is possible that participant engagement in this intervention may depend on their level of comfort with a VR environment, their readiness to address problematic components of their trauma, or challenges associated with forming a meaningful therapeutic alliance with the 3MDR clinician. Although this is not expected to be the case, and the research team has incorporated proactive steps into the protocol and procedures (ie, introductory session to familiarize the participant with the CAREN system and to develop rapport with their clinician), assessing individual readiness will be more challenging. Fourth, it is not possible to stratify randomization by gender given the limited number of women in combat roles; this is an important limitation to address in future work. Moreover, it is not possible to standardize the treatment as usual that the participants receive, which adds some variability that cannot be controlled for. Finally, as a characteristic of many rehabilitation intervention trials, participants blinding themselves to the intervention, or having a true placebo group, is not possible with current approaches. As assessors and health care professionals will work closely with the participants in the same hospital setting, blinding of these personnel is also unlikely.

### Conclusion

This RCT will provide a useful evaluation of 3MDR as a potentially effective treatment for combat-related TR-PTSD among CAF-SMs and veterans. With military members and veterans experiencing reduced effectiveness with traditional “gold-standard” PTSD interventions, there is an immediate need for creation of an effective manualized combat-related TR-PTSD intervention. This 3MDR study protocol honors previously published international studies while incorporating concepts of resilience, moral injury, technology acceptance and usability, multiomic biomarker data, machine learning, and the 3MDR clinician and operator experience. The addition of novel technologies such as eye tracking and EEG, as well as data analysis techniques such as PLS-SEM and machine learning will push the knowledge of 3MDR effectiveness as well as elucidating by what mechanisms this may be caused. It is hoped that the results of this 3MDR study will contribute to future evidence-based research and clinical accessibility to the intervention as indicated for multiple trauma-affected populations.

## Appendix

Multimedia Appendix 1 Details of the biomarker collection protocol.

Multimedia Appendix 2 Description of the outcome measures planned for this study.

## Abbreviations

3MDR: Modular Motion-assisted Memory Desensitization and Reconsolidation
CAF: Canadian Armed Forces
CAF-SMs: Canadian Armed Forces service members
CAREN: Computer Assisted Rehabilitation ENvironment
DSM-5: Diagnostic and Statistical Manual of Mental Disorders, Fifth Edition
EEG: electroencephalography
EMDR: eye movement desensitization and reconsolidation
PLS: partial least squares
PLSDA: partial least squares-discriminant analysis
PTSD: posttraumatic stress disorder
RCT: randomized controlled trial
TR-PTSD: treatment-resistant posttraumatic stress disorder
UTAUT: unified theory of acceptance and usability of technology
VR: virtual reality
