# Supplementary material for: Virtual Reality–Based Treatment for Military Members and Veterans With Combat-Related Posttraumatic Stress Disorder: Protocol for a Multimodular Motion-Assisted Memory Desensitization and Reconsolidation Randomized Controlled Trial
Source: JMIR Res Protoc. 2020 Oct 29;9(10):e20620. doi: 10.2196/20620 (PMC7661230; doi:10.2196/20620)
Supplement: Multimedia Appendix 1 [file resprot_v9i10e20620_app1.docx]

## **Multimedia Appendix 1: Biological Sample Collection and Multiomic Analyses**

Current evidence suggests that PTSD originates from a complex interplay between constellations of genes, transcripts, and proteins, environmental trauma exposures, and other biological risk / resilience factors*.* To investigate the disease state and response to treatment, this study will utilize a suite of integrative multi-omics technologies – applying genomics, epigenomics, transcriptomics, and proteomics to generate a comprehensive multi-dimensional, systems-level data and advanced biocomputational methods that allow efficient integration of these complex

multi-omics data sets for systems biology analysis.

### Blood Samples

Peripheral blood samples totalling 30 ml (serum, plasma, and PAXGene RNA) will be collected from participants by a trained phlebotomist at each of the designated time points (T1, T2.1, T2.3, T2.6, T3, T4, T5, T6) for integrative multi-omics analyses. Pre-/post-treatment blood samples would be collected on weeks 4 (T2.1) and 9 (T2.6) of the 3MDR intervention. Resting samples would be collected on weeks 3, 10, and 1, 3, and 6 month follow up (T1, T3, T4, T5, T6). A post blood sample would be drawn on week 6 (T2.4). Serum, plasma, and PAXGene RNA samples will be processed and stored at -80°C until analysis.

At T2.1 and T2.6, both pre and post 3MDR sessions, 1 mL of sodium heparin blood is added to the standardized TruCulture^®^ tubes (Myriad RBM, Austin, TX, USA) with pre-filled cell culture medium of immunological stimulants lipopolysaccharide (LPS) (E. *coli*, O55:B5) (100 ng/mL) plus Staphylococcus aureus enterotoxin-B (SEB) (100 ng/mL) and a control (null), incubating at 37°C overnight. After 24 hours, the supernatant is separated by a plunger and stored at -80°C.

At T_1_ and T_3,_ 50 mL of K_2_ EDTA blood was added on an anti-oxidant-treated filter paper card and sent for Omega-3 Index Complete Test^®^ (OmegaQuant Analytics, LLC, Sioux Falls, SD, USA). The Omega-3 index includes the percentage of eicosapentaenoic acid (EPA) and docosahexaenoic acid (DHA) in the red blood cell membranes, which is indicative of the amount of omega-3 in the body. In addition, it provides the Omega-6/Omega-3 ratio by analyzing 7 omega-6 fatty acids and 4 omega-3 fatty acids from the whole blood.

### Saliva Samples

### To characterize salivary RNA profiles, saliva samples are collected using the passive drooling method into the Oragene RNA saliva collection kit (DNAgenotek, Ottawa, ON, Canada) at baseline (T1), before (pre) and immediately after (post) 3MDR sessions at T2.1 and T2.6, post 3MDR sessions at T2.3 and T2.4, at 1-week follow up (T3), and 1-, 3-, 6-month follow ups (T4, T5, T6). Samples will then be stored at -80°C until for genomic and epigenomic analyses.

All samples will be securely stored in a locked refrigerator-freezer until the target sample size is reached and data collection is terminated. De-identified batched specimens will be delivered to Defense Research and Development Canada (DRDC) Toronto Research Centre for advanced proteomic and genomic analyses.

### Proteomic Analysis

Plasma samples will be analyzed for immune-related biomarkers with microfluidic cartridge based multiplex assays using the ELLA instrument (ProteinSimple, San Jose, CA, USA). These multiplexed immunoassay panels will assess a large number of cytokines, chemokines, and receptors [interleukin (IL)-1β, -2, -4, -5, -6, -10, -12, -13, -15, -16, -17, IL-1 receptor antagonist (IL-1RA), IL-6 receptor (IL-6R),tumor necrosis factor (TNF)-α/β, TNF-α receptors (TNFR)-I/II, interferon (IFN)-γ],Eotaxin, IFN-γ-induced protein (IP)-10, granulocyte-macrophage colony stimulating factor (GM-CSF), monocyte chemoattractant protein (MCP)-1,-4, macrophage-derived chemokine (MDC), macrophage inflammatory proteins (MIP)-1β]; acute phase proteins [c-reactive protein (CRP), serum amyloid A (SAA)]; endothelial activation/injury molecules (E-Selectin, P-Selectin, vascular cell adhesion molecule (VCAM)-1, intercellular adhesion molecule (ICAM)-1, ICAM-3, syndecan-1]; oxidative/lytic enzymes [matrix metalloproteinases (MMP)-1,2,3,9,10, myeloperoxidase (MPO)]; neurotrophins [brain-derived neurotrophic factor (BDNF), vascular endothelial growth factor (VEGF), neuropeptide-Y]; neuro-injury markers [glial fibrillary acid protein (GFAP), S100b, neuron-specific enolase (NSE)] involved in pathways hypothesized to be involved in the pathophysiology of psychiatric disorders.

Neuroendocrine hormonal responses to treatment will be assessed by measuring serum cortisol, dehydroepiandrosterone sulfate (DHEA-S), corticotropin-releasing hormone (CRH) and plasma adrenocorticortropic hormone (ACTH) levels by IMMULITE 1000 (Siemens Healthineers).

### Gene Expression Profiling and MicroRNA Analysis

PAXgene Blood RNA and saliva RNA samples will be isolated with RNA extraction kits according to manufacturer’s protocol. After isolation, whole blood and saliva gene expression will be quantified using a patented Multi-Omic Sequencing Platform, TruGen-1, which is developed by TruGenomix (TruGenomix, Rockville, MD, USA). The Tru-Gen-1 test utilizes latest next-generation sequencing technology, Illumina’s custom RNA AmpliSeq gene expression panel, which comprises a highly-specialized and curated panel of genes that are associated with PTSD, including the 3 key PTSD gene biomarkers, FK506-binding protein 51 (FKBP5), signal transducer and activator of transcription (STAT5B) and nuclear factor I/A (NFIA).

### Biological Statistical Analysis

The proposed statistical and bioinformatics analyses for peripheral blood proteomic and genomic molecular profiles will be performed using a combination of univariate and multivariate modeling approaches to address both hypothesis-driven investigations, along with a variety of analytics pipelines with supervised and unsupervised techniques that will allow exploratory analyses to identify subgroups of patients with shared symptoms and/or biological features/molecular biosignatures that may have clinical outcome relevance. All analyses will be performed using Linear Model Fitting with empirical Bayesian tests in R (RStudio, version 1.1.463, Boston, United States).
