# Supplementary material for: Virtual Reality–Based Treatment for Military Members and Veterans With Combat-Related Posttraumatic Stress Disorder: Protocol for a Multimodular Motion-Assisted Memory Desensitization and Reconsolidation Randomized Controlled Trial
Source: JMIR Res Protoc. 2020 Oct 29;9(10):e20620. doi: 10.2196/20620 (PMC7661230; doi:10.2196/20620)
Supplement: Multimedia Appendix 2 [file resprot_v9i10e20620_app2.docx]

## Multimedia Appendix 2: Outcome Measures

### Demographics Questionnaire

Demographic information will include gender, age, ethnicity, marital status, military branch (i.e., army, navy, or air force) and occupation.

### PTSD Symptoms

#### CAPS5

The primary outcome will be symptoms of PTSD measured by the Clinician Administered PTSD Scale for DSM-5 (CAPS5). The CAPS5 is a 29-item structured interview for assessing PTSD diagnostic status and symptom severity. The CAPS is the gold standard in PTSD assessment and can be used to make a current (past month) or lifetime diagnosis of PTSD or to assess symptoms over the past week. Items correspond to the DSM5 criteria for PTSD. Previous versions of the CAPS have excellent reliability and excellent convergent and discriminant validity, diagnostic utility, and sensitivity to clinical change [1].

#### PCL-5

The *PTSD Checklist* (PCL-5) will act as a secondary outcome measure at each 3MDR session. It is used to measure PTSD symptom severity by self-report. The PCL-5 is a 17-item questionnaire about symptoms in relation to an identified “stressful experience” [2,3]. This questionnaire corresponds to the DSM-IV symptom criteria for PTSD. Each symptom can be rated on a 0-4 scale. This approach has been used before and has strong reliability and validity [2,3].

#### PABQ

The *Posttraumatic Avoidance Behavior Questionnaire* (PABQ) will be used to identify explicit avoidance behavior. The PABQ is a 25-item self-report inventory, consisting of seven subscales measuring typical situations and activities that PTSD patients avoid [4]. Larger scores indicate increased avoidance behaviour [4].

#### PDEQ

The Dissociative Experiences Questionnaire (PDEQ) is a 10-item test that measures the extent of dissociation at the time of the traumatic event, and in the minutes and hours that followed [5]. Studies suggest that dissociation increases the risk of developing PTSD [5]. PDEQ administration and scoring takes under 5 minutes each. All items are scored from 1 (not at all true) to 5 (extremely true) and the total score is the sum of all items [5]. A score above 15 is indicative of significant dissociation [5].

### DER-SF-18

#### The Difficulties in Emotion Regulation Scale (DERS) is a 36 item self-report measure that aims to assess emotion dysregulation. It has been translated into several languages; short forms have been developed including the 18 item DERS-18 [6]. Items are rated on a scale of 1 (“*almost never [0–10%]*”) to 5 (“*almost always [91–100%]*”). Higher scores indicate more difficulty in emotion regulation [7].

#### Neurocognitive Functioning

The BrainFx Screen is a 30-minute assessment - it is the more comprehensive assessment designed to measure the functional impact of mild to moderate brain injury or disorder. BrainFx measures neurofunction via tablet through interactive and engaging performance activities. Assessments help to measure and evaluate cognitive functional skills, including mood, social, behavioral, fine motor and balance effects [8]. This assessment has been demonstrated to have validity and reliability that rivals gold standard cognitive screens [9].

#### Subjective Units of Distress

The *Subjective Units of Distress* Scale (SUDS) is used to rate levels of distress between 0-10, with 0 being not disturbing and 10 being extremely disturbing [10].

#### Life Events Checklist

The Life Events Checklist for *DSM-5* (LEC-5) is a self-report measure designed to screen for potentially traumatic events in a respondent's lifetime. The LEC-5 assesses exposure to 16 events known to potentially result in PTSD or distress and includes one additional item assessing any other extraordinarily stressful event not captured in the first 16 items. [11]. This tool is intended to gather information about the potentially traumatic experiences a person has experienced. There is no formal scoring protocol or interpretation other than identifying whether a person has experienced one or more of the events listed [12]. Respondents indicate varying levels of exposure to each type of potentially traumatic event included on a 6-point nominal scale, and respondents may endorse multiple levels of exposure to the same trauma type [11,12]. The LEC-5 does not yield a total score or composite score [12].

## Moral Injury

## MISS-M-SF

The Military Injury Symptom Scale – Military Short Form (MISS-M-SF) is a reliable and valid measure of MI symptoms that can be used to screen for MI and monitor response to treatment in veterans and active duty military with, or without, diagnosed PTSD [13]. The possible range of scores is from 10 to 100. The total score is an indication of functional impairment caused by moral injury or distress [13].

## Depression

### PHQ-9

The PHQ-9 questionnaire will be used to measure the severity of depression. The PHQ-9 incorporates DSM-IV depression diagnostic criteria into a 9-item, self-report questionnaire [14].  Responses represent the frequency of symptoms in the past two weeks and each symptom can be rated a 0-3 scale [14]. A score between 5-9 indicates mild depression; 10-14 indicates moderate depression; 15-19 indicates moderately severe depression; and 20-27 indicates severe depression [14].

## Anxiety

### GAD-7

The *Generalized Anxiety Disorder* (GAD-7) questionnaire will be used to measure the severity of anxiety. This is a self-report scale and consists of 7 items and responses represent the frequency of symptoms in the two past weeks and are given on a 0-3 scale [15]. Scores between 5-9 indicate mild anxiety; 10-14 indicate moderate anxiety; and 15-21 indicate severe anxiety [15].

## Social Functioning

### OQ-45

The Outcome Questionnaire–45 is a self-report inventory measuring social functioning. It consists of 45 items which are rated on a 5-point scale and reflect three domains: symptomatic distress (SD), interpersonal relationships (IR) and social role (SR) [16]. A total score of 63 or more indicates symptoms of clinical significance and a difference of 14 points or more (between sessions) indicates a significant change in symptoms (OQ45) [16].

## Alcohol Use

### AUDIT

The Alcohol Use Disorders Identification Test (AUDIT) is an alcohol self-report, 10-item questionnaire [17]. The AUDIT questionnaire helps identify persons who are hazardous drinkers or have active alcohol use disorders [17]. A score of 8 or more is considered to indicate hazardous or harmful alcohol use [17].

## Quality of Life

### EQ5D-5L

The EQ5D-5L is a widely used instrument in health economic analysis and is an appropriate measure for health-related quality of life [18]. The questionnaire provides a simple descriptive profile, which translates to a single utility score for health status. The first part of the instrument identifies the extent of perceived problems across five levels in five life dimensions: mobility; self-care; usual activities; pain and discomfort; and anxiety and depression [17]. The responses to each of the five questions are used to generate a utility score for self-rated health status on a 0-1 scale, where 0 represents the worst possible health state and 1 the best possible health state [18]. The second part is a visual analogue scale, which allows the responder to indicate their current health status on a 0-100 scale [18].

## Resilience

The Connor-Davidson Resilience Scale is a tool utilized to measure perceived resilience within 17 domains. The tool consists of a 25-item scale within these domains. This tool has been studied extensively and has demonstrated to be valid and reliable when utilized with survivors of various traumas and PTSD [19].

## 3MDR Specific Domains

### 3MDR-Q

A 3MDR questionnaire (3MDR-Q) has been designed to assess presence, working memory load, cognitive avoidance and change after each 3MDR session. It contains 16 questions which can be rated on a 5-point Likert scale.

### CSQ-8

The *Client Satisfaction Questionnaire* (CSQ-8) was selected to measure patient treatment satisfaction [20]. The CSQ-8 consists of 8 questions and each question is rated *Poor to Excellent* on a 1-4 scale [20].

## Technology Acceptance and Usability

### UTAUT Questionnaires

The unified theory of acceptance and use of technology (UTAUT) is a [technology acceptance model](https://en.wikipedia.org/wiki/Technology_acceptance_model) [21]. The UTAUT aims to explain user intentions to use an [information system](https://en.wikipedia.org/wiki/Information_systems) [information system](https://en.wikipedia.org/wiki/Information_systems) and subsequent usage behavior [21]. model addresses the perceived expectations of technological acceptance of new technology in 5 domains: 1) Performance Expectancy (PE), 2) Effort Expectancy (EE), 3) [social influence](https://en.wikipedia.org/wiki/Social_influence) (SI), 4) facilitating conditions (FC) and 5) Behavioural Intentions (BI) which have a direct impact upon use behavior [21]. UTAUT Questionnaire for a. Patients, b. Clinicians and Operators.

The UTAUT Model guided the creation of an outcome measure specific to the patient’s perception of usability and effectiveness of 3MDR with the CAREN. Two 15 question UTAUT survey versions were developed with 3 questions per domain. Version I (T0, future tense) was completed prior to exposure to the CAREN and 3MDR for all user groups. Version II (T1, past tense) was administered after completion of all 6 3MDR sessions. The 15 questions outcome measure is based on a Likert Scale with a score of 0 -7 assigned to each question with 0 being “strongly disagree” and 7 being “strongly agree”. The maximum score is 105. The user is considered a variable in this model and outcome measure. If there is a change of 5% or more, then it is an indication that the user has an effect. The results of the outcome measure will be compared to the qualitative results from the semi-structured interview.

## 3MDR Clinicians and Operator Specific Outcome Measures

### PPS

The Perceived Stress Scale (PSS) is a classic 10-item stress assessment instrument. The tool, while originally developed in 1983, remains a popular choice for helping us understand how different situations affect our feelings and our perceived stress [22]. This scale asks about the respondent’s feelings and thoughts during the last month. In each case, the respondent is asked to indicate how often they felt or thought. Respondents rate each of the ten questions from 0-never, 1-almost never, 2-sometimes, 3- fairly often, 4- very often [22]. Overall scores range from 0-40 with scores ranging from 0-13 being considered low stress, 14-26 moderate stress, and 27-40 high perceived stress [22].

### ProQOL Version 5

The Professional Quality of Life scale ProQOL is a 30-item self-rating tool that assesses compassion satisfaction and compassion fatigue associated with helping others [23]. Respondents select a response that honestly reflects how frequently they have experienced positive and negative experiences as a helper in the 30 days prior (rating scale: 1=never, 2=rarely, 3=sometimes, 4=often, 5= very often) [23].

### Secondary Traumatic Stress Scale

This 17-item scale provides a list of statements made by the participant who have been impacted by their work with traumatized clients [24]. The scale includes 3 subscales: intrusion, avoidance, and arousal, all of which are combined to provide a total score [24]. Respondents are to indicate how frequently the statement was true for them as a person in a helping relationship in the past 7 days. Statements are rated 1=never, 2=rarely, 3= occasionally, 4=often, 5=very often [24].

### References

1. Blake DD, Weathers F.W, Nagy LM, Kaloupek DG, Gusman FD, Charney DS, Keane TM. (1995). The development of a clinician-administered PTSD scale. *Journal of Traumatic Stress, 1*995;8:75-90. doi: 10.1002/jts.2490080106.
2. Renshaw KD, CaskaCM. Relationship Distress in Partners of Combat Veterans: The role of Partners’ Perceptions of Posttraumatic Stress Symptoms. *Behavior Therapy,* 2012;43:416-426.
3. Renshaw KD, Rodrigues CS, Jones DH. Psychological symptoms and marital satisfaction in spouses of Operation Iraqi Freedom veterans: Relationships with spouses’ perceptions of veterans’ experiences and symptoms. *Journal of Family Psychology,* 2008;22:586–594. doi: [10.1037/0893-3200.22.3.586](https://www.researchgate.net/deref/http%3A%2F%2Fdx.doi.org%2F10.1037%2F0893-3200.22.3.586?_sg%5B0%5D=5DDvk5t6sYgAb9ZSj0tBR38HyEvURpCF_8_Z98cowZgzD32Or0MRlbx8To3Fgm4JR20jJTSfTP7Bf3g9qMr9SM9OlA.4n-vUb14_WZFC46rORtH6kpGFhYv0wNz7nLBA9dxeFUMblAqxTyJr8Sp5IVxZqexLCGUQkwN2O37VqkRgNjjJA)
4. V[an Minnen A, Hagenaars MA. Avoidance behaviour of patients with posttraumatic stress disorder: Initial develop-ment of a questionnaire, psychometric properties and treat-ment sensitivity. *Journal of Behavior Therapy and* *Experimental Psychiatry*, 2008;41:191-198](https://www.google.com/search?rlz=1C5CHFA_enCA880CA880&q=Van+Minnen,+A.,+%26+Hagenaars,+M.+A.+(2010).+Avoidance+behaviour+of+patients+with+posttraumatic+stress+disorder.+Initial+develop-ment+of+a+questionnaire,+psychometric+properties+and+treat-ment+sensitivity.Journal+of+Behavior+Therapy+and+Experimental+Psychiatry,41,+191?198&spell=1&sa=X&ved=2ahUKEwiM6N6X0IrqAhXXrJ4KHfL-ChIQBSgAegQIDBAm). doi:10.1016/j.jbtep.2010.01.002
5. Birmes P, Brunet A, Carreras D, et al. The predictive power of peritraumatic dissociation and acute stress symptoms for posttraumatic stress symptoms: a three-month prospective study. *Am J Psychiatry*. 2003;160(7):1337-1339. doi:10.1176/appi.ajp.160.7.1337.
6. Victor, S. E., & Klonsky, E. D. (2016). Validation of a brief version of the Difficulties in Emotion Regulation Scale (DERS-18) in five samples. Journal of Psychopathology and Behavioral Assessment, in press. doi: [10.1007/s10862-016-9547-9](https://www.researchgate.net/deref/http%3A%2F%2Fdx.doi.org%2F10.1007%2Fs10862-016-9547-9?_sg%5B0%5D=FmgNu0LeRSQvcIPAgQlI0eTlwa4vSQ3IZxyva_gmpM2JWnnN9hq8ugyb9gMoWq-tPjpImsFVPFrdtLP4NXCz5ZPskA.IEnlKrS-BsHN63O0JVgf0S3BA6iIptEWUEJIDRQiT94QWN9CL-ZhqiANg_KBAZ11Yo5cPMHYIfgMOEwr9wuu9w)
7. Gratz K, Roemer L. Multidimensional Assessment of Emotion Regulation and Dysregulation: Development, Factor Structure, and Initial Validation of the Difficulties in Emotion Regulation Scale*. Journal of Psychopathology and Behavioral Assessment*. 2004;26(1):41-54. doi: 10.1023/B:JOBA.0000007455.08539.94.
8. Milner T, Condello H. BrainFX Screen. <https://www.brainfx.com/brainfx-screen/> Updated 2020. Accessed September 18, 2019.
9. Searles CM, Farnsworth J, Jubenville C, Kang M, Ragan B. Test-retest reliability of the BrainFx 360 performance assessment. *Athletic Training & Sports Health Care*, 2019;11(4):183-191. doi: [10.3928/01913913-20181005-01](https://www.researchgate.net/deref/http%3A%2F%2Fdx.doi.org%2F10.3928%2F01913913-20181005-01?_sg%5B0%5D=aN7zpZrqpl-PKRP8vcHACIyJ4Vh4Gns49ZtJhr4l0tRBYjP-59ifuU5Q-vJucRbQw8_wtfzmt4-ZGK-Y3VrP02gbzA.IVZPrFnspXri3lBZaB2biqMkuyViTFy3mlaqJHngGs7QVltk8SkKN8CjpQl7rGakqG2eUHhtAIfhnEb2N-smqg)
10. Wolpe J. (1969), *The Practice of Behavior Therapy*, New York, NY: Pergamon Press; 1969.
11. Gray M, Litz B, Hsu J, Lombardo T. Psychometric properties of the Life Events Checklist. *Assessment,* 2004;11:330-341. doi: 10.1177/1073191104269954
12. Weathers, F. W., Litz, B. T., Keane, T. M., Palmieri, P. A., Marx, B. P., & Schnurr, P. P. (2013). The PTSD Checklist for DSM-5 (PCL-5) – LEC-5 and Extended Criterion A [Measurement instrument]. http://www.ptsd.va.gov/ Updated 2019. Accessed November 18, 2018.
13. Koenig H, Ames D, Youssef NA, Oliver JP, Volk F, Teng EJ, Haynes K, Erickson, ZD, Arnold I, O’Garo K, Pearce Screening for Moral Injury: The Moral Injury Symptom Scale – Military Version Short Form, Military Medicine *2018;183(11):*e659–e665. doi: [10.1093/milmed/usy017](https://doi.org/10.1093/milmed/usy017).
14. Kroenke K, Spintzer RL, Williams JB. The PHQ-9: validity of a brief depression severity measure. Journal of General Internal Medicine 2001;16(9):606-613. doi: 10.1046/j.1525-1497.2001.016009606.x.
15. Spitzer RL, Kroenke K, Williams JB, Löwe B. A brief measure for assessing generalized anxiety disorder: the GAD-7. *Arch Intern Med*. 2006;166(10):1092-1097. doi:10.1001/archinte.166.10.1092
16. Lambert MJ, Hansen NB, Umphress V, Lunnen K, Okiishi J, Burlingame G, Reisinger CW. Administration and scoring manual for the Outcome Questionnaire (OQ45.2) Wilmington, DE: American Professional Credentialing Services; 1996.
17. Bradley KA, Bush KR, Epler AJ, et al. (2003). Two brief alcohol-screening tests From the Alcohol Use Disorders Identification Test (AUDIT): Validation in a female Veterans Affairs patient population. Arch Intern Med. 163:821-9. doi: 10.1001/archinte.163.7.821.
18. Herdman M, Gudex C, Lloyd A, Janssen M, Kind P, Parkin D, Bonsel G, Badia X. [Development and preliminary testing of the new five-level version of EQ-5D (EQ-5D-5L)](https://eq-5dpublications.euroqol.org/details?id=152_4002&nosearchform=true). Qual Life Res, 2011, 20;10:1727-1736. doi: [10.1007/s11136-011-9903-x](https://dx.doi.org/10.1007%2Fs11136-011-9903-x)
19. Connor KM, Davidson JRT. Development of a new resilience scale: the Connor-Davidson Resilience Scale (CD-RISC). Depression and Anxiety, 2003:18:71-82. doi: 10.1002/da.10113.
20. Larsen DL, Attkisson CC, Hargreaves WA, Nguyen TD. Assessment of client/patient satisfaction: development of a general scale. *Eval Program Plann*.1979;2(3):197-207. doi:10.1016/0149-7189(79)90094-6.
21. Venkatesh V, Morris M, Davis G, Davis F. User acceptance of information technology: toward a unified view. Mis Quart 2003;27: 425–78. doi:10.2307/30036540.
22. Cohen S, Williamson G. Perceived Stress in a Probability Sample of the United States. Spacapan, S. and Oskamp, S. (Eds.) The Social Psychology of Health. Newbury Park, CA: Sage; 1988. doi: 10.13072/midss.461.
23. Stamm BH. The Secondary Effects of Helping Others: A Comprehensive Bibliography of 2,017 Scholarly Publications Using the Terms Compassion Fatigue, Compassion Satisfaction, Secondary Traumatic Stress, Vicarious Traumatization, Vicarious Transformation and ProQOL. http://[www.proqol.org](http://www.proqol.org/). Published January 16, 2016. Accessed June 18, 2020.
24. Bride B. The prevalence of secondary traumatic stress among social workers. *Social Work*, 2007;52(1):63. doi: [10.1093/sw/52.1.63](https://www.researchgate.net/deref/http%3A%2F%2Fdx.doi.org%2F10.1093%2Fsw%2F52.1.63?_sg%5B0%5D=rpSQ_Tk3iSLCleqq-uOx6sHq7akZF8d7o19rANYf-wCstFCAvgaaj8YNDJt8Oyxs2rKB45gPP2MzkurOspCWft_saA.-hJD88ZHpUEdpFaLUxZiLdC2kYXIJds0ksvofhDGlfkGkQnFzmuC8lqJUADEME7VUXsJfWi45cP3aJ_ra2swqA).
